# Excessive Paranasal Sinuses and Mastoid Aeration as a Possible Cause of Chronic Headache

**DOI:** 10.1155/2013/836064

**Published:** 2013-12-04

**Authors:** Panagiotis Kousoulis, Jiannis Hajiioannou, Vassiliki Florou, Dimitrios Kretzas, George Korres

**Affiliations:** ^1^Department of Otorhinolaryngology, General Hospital of Nikea-Piraeus, Greece; ^2^Department of Otorhinolaryngology, “Attikon” University Hospital of Athens, Greece

## Abstract

The objective of this case report is to present a patient with chronic headache who was diagnosed with excessive aeration of all paranasal sinuses and mastoid air cells using computed tomography imaging. The volume and linear measurements of all of the cavities revealed values greater than the greatest values reported in the literature. To date, this is the second reported case of excessive enlargement of all paranasal sinuses and the first which includes the enlargement of the mastoid air cells. No surgical intervention was required for the patient, but in similar cases, with more severe symptoms, surgical treatment is a challenge for the surgeon and may mandate a multidisciplinary approach.

## 1. Introduction

Excessive enlargement of the paranasal sinuses is a rare entity with an uncertain aetiology. In the medical literature, it has been described with many terms including hypersinus, pneumocele, pneumatocoele, sinus ectasia, hyperpneumatization, and pneumosinus dilatans. It usually affects the frontal sinus, although any sinus can be pathologically enlarged. We present a case of a patient with chronic headache, diagnosed with excessive aeration of all paranasal sinuses, together with atypical mastoid pneumatization. To the authors' knowledge, this has not been previously reported in the literature.

## 2. Case Presentation

A 38-year-old woman was referred for evaluation to the outpatient otorhinolaryngology clinic by the neurology department. The patient complained of intermittent episodes of moderate, nonthrobbing, and severe pressure-like headache since early adulthood. The headache was typically located at the anterior part of the cranium, mainly over the frontal, the anterior parietal, and temporal area. The symptoms usually lasted for hours and were sufficiently relieved by common analgesics such as paracetamol and nonsteroid anti-inflammatory drugs. The pain was not accompanied by facial swelling and was not related to head position (bending forward, lying down, or sitting up). Palpation of the face and anterior cranium did not reproduce the symptoms and no palpable or visually evident anatomic deformity was noted.

Neurologic and ophthalmologic examinations were unremarkable. Routine laboratory parameters were within normal range. Nasal endoscopic examination did not reveal pathologic findings. The nasal septum was slightly deviated while the maxillary and sphenoid ostia appeared patent. Although the frontal sinus ostium could not be visualized endoscopically on either side, no mucosal abnormalities were apparent at the area of the frontal sinus outflow tract.

**Figure 1:**
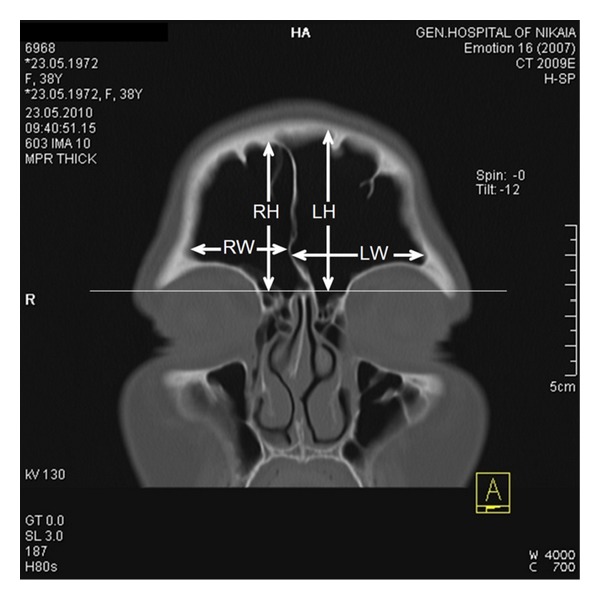
Linear measurements of frontal sinus in the coronal plane (RW: width of the right sinus, LW: width of the left sinus, RH: height of the right sinus, and LH: height of the left sinus).

**Figure 2:**
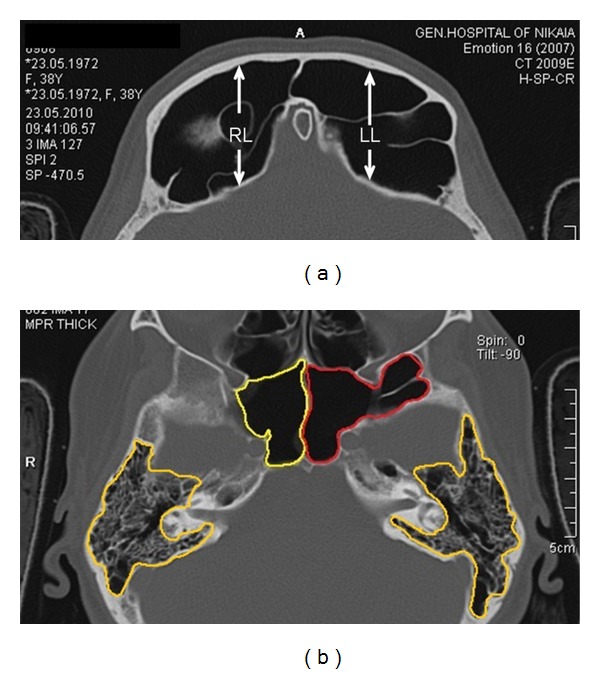
(a) Linear measurements of frontal sinus in the axial plane (RL: anteroposterior length of the right sinus and LL: anteroposterior length of the left sinus). (b) Area calculation of the sphenoid sinuses and the mastoid air cells in the axial plane.

**Figure 3:**
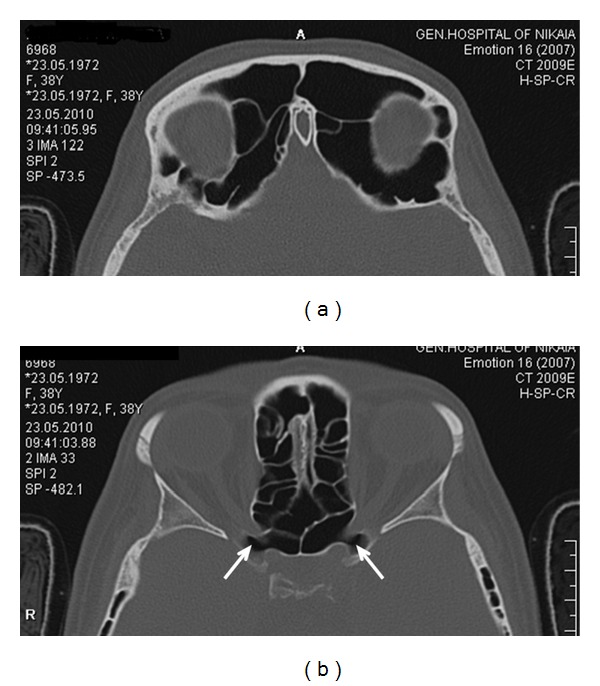
(a) The frontal sinus enchroaching the orbit. (b) The optic nerves are exposed (arrows) in the superior part of the sphenoid sinus.

**Figure 4:**
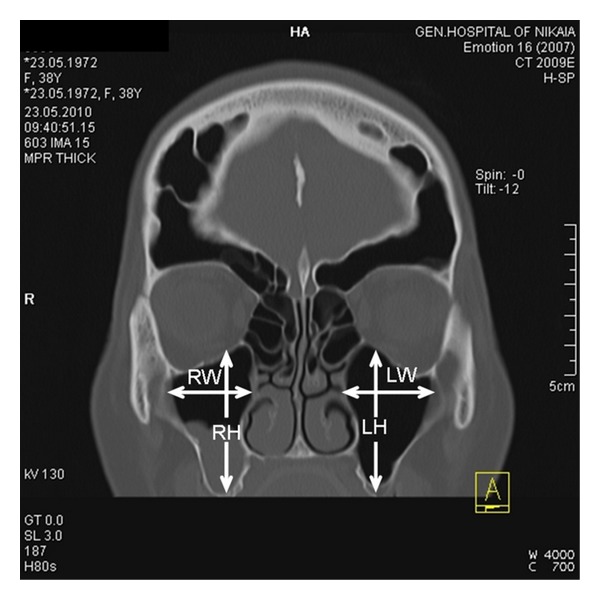
The frontal lobe looks smaller in size due to the enlargement of the frontal sinus. Linear measurements of maxillary sinus (RW: width of the right sinus, LW: width of the left sinus, RH: height of the right sinus, and LH: height of the left sinus).

A high resolution CT scan revealed that all the paranasal sinuses and especially the frontal sinuses were excessively enlarged. Similarly, the mastoid air cells showed bilaterally excessive pneumatization. No signs of bony erosion or thinning were evident in the CT scan. The frontal sinus extended posteriorly and laterally, encroaching the ophthalmic bulb. The frontal lobe appeared to be smaller in size, due to the posterior enlargement of the frontal sinus. No other abnormalities were found, apart from a retention cyst in the right maxillary sinus (Figures 1–4).

Measurements of the linear dimensions of the frontal sinuses were performed according to the procedure described by Tatlisumak et al. [1]. The width, height, and anteroposterior length of each frontal sinus and the total width of both sinuses were calculated (Figures 1 and 2).

Volumes of the frontal, sphenoid and maxillary sinuses, and mastoid air cells were calculated according to the method used by Karakas and Kavakli [2] and Pondé et al. [3]. The area of each sinus was measured in every axial CT image (Figure 2) and multiplied by the slice thickness. The sum of all measurements was equal to the volume of the sinus.

The type of extensive maxillary sinus pneumatization (EMSP) was determined according to the classification of Kalavagunta and Reddy [4] comparing the height and width of the maxillary sinus with the diameter of the ipsilateral ophthalmic bulb (Figure 4).

## 3. Discussion

Terms like pneumosinus dilatans, pneumatocele, hyperpneumatization, sinus ectasia, sinus hypertrophy, and aerocele have been used in the literature to describe a hyperpneumatized paranasal sinus. An intuitive and simplified classification has been suggested by Urken et al. describing only three types of sinus hyperpneumatization [5]. Hypersinus refers to an enlargement of a well-aerated frontal sinus which develops within the normal boundaries of the frontal bone. Pneumosinus dilatans differentiates from Hypersinus because the sinus abnormally extends either anteriorly, causing frontal bossing, or posteriorly and laterally, displacing the adjacent anatomic structures like the ophthalmic bulb and the frontal lobe. A pneumatocele is the third type of hyperpneumatization which is characterized by focal erosion or generalized thinning of the bony walls, while in the first two types the sinus walls are normal. Although this classification was developed for the description of frontal sinus anatomic variations, it has been used in the literature for all paranasal sinuses. According to this classification, the present case is described as “pneumosinus dilatans of the frontal sinuses” since the bony walls were intact and the frontal sinus expanded posteriorly and laterally, causing an enlargement of the frontal bone and a reduction in the frontal lobe size. Additionally, the patient was diagnosed with “Hypersinus” of the sphenoid and maxillary sinuses and “Hyperaeration” of the mastoid air cells.

It is widely accepted that headache is one of the symptoms of sinonasal diseases. The term “sinus headache” refers to a secondary headache associated with sinusitis that occurs when the sinuses become congested and obstructed because of paranasal sinus pathology. “Sinus headache” is characterized by episodes of pain over the sinus area of the face or around the eyes and is typically accompanied with nasal congestion and rhinorrhea. Cases with pneumosinus dilatans complicated with sinus pathology and presenting with sinus headache have been reported in the literature [6]. The present case in which headache was the first and only symptom of the patient does not fall within the diagnostic criteria of “sinus headache” due to the absence of sinus obstruction and mucosal pathology. To the authors' knowledge, this is the second reported case of excessively aerated frontal sinus that presents with symptoms of headache but without signs and symptoms of sinus disease [7].

The characteristics of the headache in the present case are consistent with tension type headache. The relief of symptoms by treating the patient with oral analgesics supports the “tension headache” diagnosis. Under this thought one may argue that the hyperaeration of the paranasal sinuses is an incidental finding and not the cause of the patient's symptoms. However, it has been suggested that hyperaeration itself may play a role in the development of headache symptoms (migraine, cluster of tension type), either by affecting the mucociliary clearance or due to disturbance of ventilation, which in turn causes vacuum headache [4].

Several papers can be found in the literature addressing the issue of sinus morphology and dimensions. Different researchers, however, have used different measuring methodologies. In the present study, the linear dimensions of the frontal sinus were measured according to Tatlisumak et al. [1] and the volumetric measurements of mastoid air cells and paranasal sinuses according to Karakas and Kavakli [2] and Pondé et al. [3]. In order to compare the findings of the present case, the results of Pondé et al. [3], Kim et al. [8], Lee et al. [9], and Kalavagunta and Reddy [4] were used. Despite the differences in methodology, all of the above-mentioned studies used CT scans of the head and paranasal sinuses for measuring dimensions and volumes. It is interesting to note that all linear (Table 1) and volumetric measurements (Table 2) in all sinuses and mastoid air cells in the present case were higher than the highest reported in the literature. The pattern of extensive maxillary sinus pneumatisation (EMSP) was “moderate - type II” according to the Kalavagunta classification [4], with both width and height of the maxillary sinus being more than 90% greater than the diameter of the ipsilateral ophthalmic bulb (Table 3). The present case is the second reported case of excessive enlargement of all paranasal sinuses [10] and the first which includes the enlargement of the mastoid air cells as well.

In the present case, the symptoms were not severe and could be alleviated by medical treatment. This is not always the case in other similar cases of sinus expansion. Complications of pneumosinus include cosmetic deformities due to the bossing of the facial bones [11], recurrent sinusitis due to obstruction of the paranasal sinus outflow tract [6], and proptosis, diplopia, or progressive blindness due to expansion towards the orbit [12]. We believe that in similar rare cases of abnormal sinus or mastoid air cells expansion, the treatment should be individualized and address the patients cardinal symptoms. An algorithm on surgical management of pneumosinus dilatans of the frontal sinus has been described by Patel et al. [11] but can be generalized for all aerated cavities. If the main symptoms are caused by obstruction of the sinuses ostia, Functional Endoscopic Sinus Surgery (FESS) may restore normal drainage. However, the surgeon should be aware that anatomic landmarks may be altered in similar cases. If a FESS procedure was to be performed in the present patient, access to the frontal sinus would be extremely difficult due to the presence of numerous frontoethmoidal air cells (Figure 4). Operating in the sphenoid would also be a challenge, since the optic nerves were exposed bilaterally in the superior part of the sinus (Figure 3). If bossing of the frontal or maxillary area causes cosmetic deformity, an open procedure with bone removal, reshaping and repositioning, with or without obliteration of the underlying sinus, can be performed. Similarly, orbital and optic nerve decompression can be achieved with an endoscopic, open or combined procedure [6].

## 4. Conclusion

We present a rare case in the literature with excessive aeration of all paranasal sinuses, along with excessive mastoid pneumatization. Although no surgical intervention was required in this patient, in similar cases, with more severe symptoms, surgical treatment is a challenge for the surgeon and may mandate a multidisciplinary approach.

## Figures and Tables

**Table 1:** Linear measurements (mm) of frontal sinus.

Frontal sinus	Patient	Lee et al. [9]	Pondé et al. [3]	Tatlisumak et al. [1]
Total width	78	50.4 ± 14.5	51.05 ± 17.14	50.45 ± 12.32
Width of left sinus	43	25.0 ± 7.7		26.05 ± 7.40
Width of right sinus	35	25.4 ± 8.0		24.37 ± 7.63
Height of left sinus	53		28.57 ± 7.36	24.70 ± 8.20
Height of right sinus	49			23.63 ± 8.33
Antero posterior depth of left sinus	40		10.16 ± 2.12	10.80 ± 4.10
Antero posterior depth of right sinus	38			10.15 ± 4.08

**Table 2:** Volume measurements (mm^3^) of frontal, maxillary, and sphenoid sinuses and mastoid air cells.

	Patient	Karakas and Kavakli [2]	Pondé et al. [3]	Kim et al. [8]
Frontal sinus				
Right	36338			
Left	38902			
** Total**	**75240**	8410 ± 4030	8028 ± 6081	**4430**
Maxillary sinus				
Right	23988	15040 ± 5200		17259
Left	24240	15970 ± 6650		17415
** Total**	**48228**	30980 ± 11410		**34675**
Sphenoid sinus				
Right	9962			
Left	11396			
** Total**	**21358**	8530 ± 4190		**10671**
Mastoid air cells				
Right	24986	9790 ± 2820		5463
Left	31160	9690 ± 2960		5911
** Total**	**56146**	19480 ± 5620		**11374**

**Table 3:** Linear measurements (mm) of maxillary sinuses and comparison with the diameter of ophthalmic bulb.

Maxillary sinus (mm)	Patient	Percentage of ophthalmic bulb diameter
Width of right sinus	31,8	126%
Height of right sinus	47,1	186%
Ophthalmic bulb right	25,2	
Width of left sinus	33,1	136%
Height of left sinus	45,2	186%
Ophthalmic bulb left	24,3	
